# Phytoplankton composition with an emphasis of Cyanobacteria and their toxins as an indicator for the ecological status of Lake Vaya (Bulgaria) – part of the Via Pontica migration route

**DOI:** 10.3897/BDJ.8.e57507

**Published:** 2020-12-16

**Authors:** Ivanka Teneva, Detelina Belkinova, Rumen Mladenov, Plamen Stoyanov, Dzhemal Moten, Diyana Basheva, Stefan Kazakov, Balik Dzhambazov

**Affiliations:** 1 Plovdiv University “Paisii Hilendarski”, Plovdiv, Bulgaria Plovdiv University “Paisii Hilendarski” Plovdiv Bulgaria; 2 Bulgarian Academy of Sciences, IBER, Sofia, Bulgaria Bulgarian Academy of Sciences, IBER Sofia Bulgaria

**Keywords:** phytoplankton composition, cyanoprokaryota, cyanotoxins, ecological indices, Lake Vaya (Burgas)

## Abstract

As producers of biomass, cyanobacteria are a major part of the phytoplankton in a large number of water basins. Due to the cyanobacterial blooms and cyanotoxins produced, these organisms are recognized as a threat and ecological risk for water bodies. Released cyanotoxins may cause death of many organisms including birds and fish. Vaya Lake is the largest natural lake in Bulgaria. It is located on the Via Pontica migration route of birds between Europe and Africa. Since 2003, the lake has been declared as a "Wetland of international importance” under the Ramsar Convention. According to the literature data from 2002-2006, the Lake is defined as highly eutrophied due to strong anthropogenic pressure, but regular monitoring of the cyanobacterial blooms and presence of cyanotoxins after this period is missing. Taking into account the importance of this unique, protected ecosystem, our aim was to make a complete ecological assessment of the present state of Lake Vaya by using the phytoplankton, with an emphasis on cyanobacterial blooms and the presence of cyanotoxins. As results of the study, we 1) characterized the phytoplankton composition qualitatively and quantitatively; 2) evaluated the ecological status of the western and eastern part of the Lake; 3) identified the potential producers of cyanotoxins; 4) observed cyanobacterial blooms and discussed the influence of macrophytes on their spread; 5) measured the concentrations of the cyanotoxins MCs, CYL and STXs in water samples from both parts of the Lake. Our results indicated the need for continued observation of cyanobacterial composition, blooming and the presence of cyanotoxins in Lake Vaya.

## Introduction

Cyanobacteria are an interesting ancient group of photosynthetic prokaryotes with a cosmopolitan distribution. They are adapted to almost all ecological niches. Cyanobacteria are found in fresh and marine waters and very often are the dominant part of the phytoplankton. Some of them are inhabitants of terrestrial environments and other extremophiles and can live in hypersaline waters, thermal springs or on the glacier's surface at the poles (Dvořák et al. 2017). As major producers of biomass, the representatives of Cyanobacteria are an integral part of the phytoplankton in a large number of water basins. In recent years, they have been reported as a co-dominant group in Bulgarian ponds together with Chlorophyta and Bacillariophyta (Teneva et al. 2018). In addition, they are recognized as a threat and ecological risk for their habitats due to "cyanobacterial blooms" that they cause and the released cyanotoxins (Huisman et al. 2018). Based on the chemical structure and effects on other organisms, cyanotoxins are classified as cytotoxins, neurotoxins, dermatotoxins or hepatotoxins (Wiegand and Pflugmacher 2005; Teneva et al. 2006; Wang et al. 2006; Du et al. 2019).

Cyanotoxins and the strains that produce them are subject to intensive research because of the dangers they pose to human health and the environmental risk they could have during the blooms' formation. The accumulation of toxins in the water bodies during "cyanobacterial blooms" also leads to the death of birds and fish and the mechanisms of action for many of these toxins are still unclear.

Lake Vaya (Burgas Lake) is the largest natural lake in Bulgaria and one of the 11 sites in Bulgaria included in the Ramsar Convention. It is a shallow liman on the Black Sea coast, situated to the west of Burgas Сity. It is located on the Via Pontica migration route and has been considered as one of the most important stations of bird migration on the Bulgarian Black Sea coast. This station is of international importance for the wintering of a significant number of waterfowl. Out of the 245 birds, 71 are included in the Red Data Book of Bulgaria, 105 are of European conservation significance and nine are world-protected. In 1989, BirdLife International announced Lake Vaya as an ornithological important place in Europe. Since 1997, the western part of the Lake has been declared as a protected area and, since 2003, the Lake has been declared as a "Wetland of international importance” under the Ramsar Convention. In 2007, Lake Vaya was included in the European Ecological Network Natura 2000 as a protected area for the conservation of wild birds.

Therefore, studies on the taxonomic composition and toxic potential of the cyanobacterial species in Lake Vaya are of great importance. These toxins may have a direct effect on the fish and aquatic plants and can cause death of many birds. On the other hand, the Lake is traditionally used for fishing. Lake Vaya is one of the many reservoirs in Bulgaria where the cyanobacterial blooms are fact, but the difference is the special status of this Lake. The Lake is a part of the bird migration route and it is a protected area. In this regard, we should place emphasis on:

1. the possible danger of poisoning and death of rare bird species and
2. the danger of the transfer of toxic cyanobacterial species by migratory birds along the migratory route.

Moreover, it is subjected to serious anthropogenic pressure and accelerated eutrophication, which are a serious prerequisite for the emergence of cyanobacterial "blooms".

Studies, related to the hydrobiological and hydrochemical parameters of Lake Vaya were performed for samples collected in the period 2004-2006 (Dimitrova et al. 2014a; Dimitrova et al. 2014b), as well as in 2017 (Agafonova 2018). HPLC analyes of water samples from Lake Vaya, collected in 2004, showed the presence of microcystin-LR (Pavlova et al. 2006). Since then, the phytoplankton data, including Cyanobacteria, are fragmented and incomplete. The Lake has not been monitored for cyanobacterial blooms and the presence of cyanotoxins after the aforementioned period.

The aim of this study was to assess the ecological status (ES) of Lake Vaya according to the Biological Quality Element (BQE: phytoplankton) with emphasis on cyanobacteria (and also cyanotoxins) in connection with the implementation of the Hungarian Lake Phytoplankton Index (HLPI). The dominant phytoplankton species, with a relative biovolume > 5% and their functional groups (FGs), have been identified. A complete taxonomic list of species is also provided. Cyanobacterial "blooms", observed during the studied period, have been analyzed and cyanobacterial species that are potential toxin producers have been identified. Additionally, the levels of cyanotoxins (microcystins and cylindrospermopsin) in the water samples from September 2018 were measured. In addition, we hypothesized the presence of a relationship between the macrophytes, cyanobacterial blooms and the amount of cyanotoxins detected at the two sampling points.

We believe that the present work enriches and deepens our knowledge about the cyanobacterial blooms, the species that cause them, the toxins that are produced and the relationships of cyanobacteria with other phytoplankton groups. The influence of cyanobacterial blooms and the cyanotoxins produced by them in the presence of macrophytes in water bodies is also taken into account.

## Material and methods

### Site description

**Figure 1.**
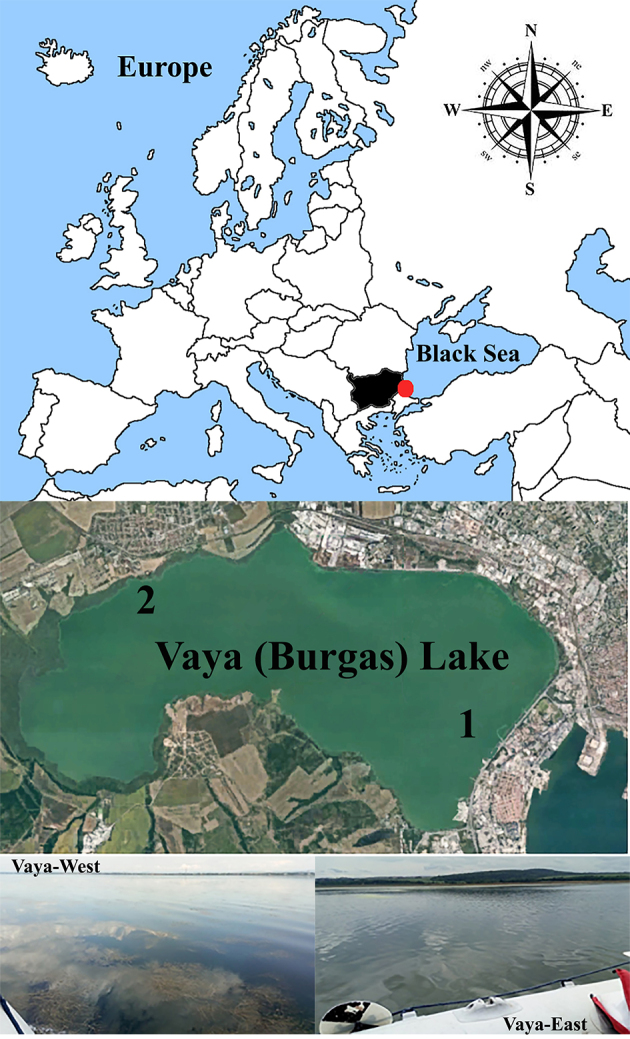
Location of the Lake Vaya (Bulgaria) and sampling stations. The red dot represents the position of the lake sampling stations : **1** – Vaya-East , **2** – Vaya-West.

Lake Burgas or Lake Vaya is the largest natural lake in Bulgaria (27 - 28 km²) which, together with Atanasovsko Lake and Mandrensko Lake, is part of the Burgas Lake Complex. It is situated in the immediate vicinity of Burgas - the second largest city on the Bulgarian Black Sea coast, which is an important industrial and transport centre (Fig. 1).

Lake Vaya is very shallow (with an average dept of 1-1.5 m), separated from the sea by a strip of sand (Kumluka) and connected to it through a narrow channel. At its southern edge, the Lake forms a shallow bay which, in the past, had a connection with Mandrensko Lake. The rivers Aytoska, Sandardere and Chukarska flow into its western part. The water in the Lake is slightly salty with significant seasonal and annual fluctuations. It is surrounded by a strip of reeds which, in the western part, forms a large and dense massif.

### Hydromorphological characteristics and physicochemical water quality analysis

The physicochemical parameters (temperature, dissolved oxygen, oxygen saturation, pH, conductivity, salinity) were measured *in situ* by using field devices of the brand WTW (Xylem Analytics Germany Sales GmbH & Co. KG, WTW, Weilheim, Germany) (Table 1).

### Sample collection and phytoplankton analysis

Phytoplankton samples were collected five times during the growing season (April-September) of 2018 from two points of Lake Vaya: East (42°29'00.6''N, 27°26'16.7''E) and West (42°30'38.6''N; 27°22'03.6''E). Station Vaya-East is located in the area of the open water surface in the eastern part of the Lake. Station Vaya-West is located in the western part of the Lake, near the reed strip and is characterized by intense growth of submerged macrophytes. Due to the polymictic nature of the Lake, the sampling was done from the subsurface layer in accordance with sampling, preservation and sample preparation standards (ISO 5667-1,3: 2012). Phytoplankton samples were preserved with formalin (4% final concentration). Along with the phytoplankton samples, additional samples were taken for measurement of chlorophyll-*a.* Its concentration was measured spectrophotometrically, following the international standard ISO 10260:1992. During the sampling in September, from the same points, we also collected water samples without fixation to analyse for presence of cyanotoxins.

The taxonomic composition of the phytoplankton (in live and preserved samples) was determined by using a light microscope Amplival (magnification up to 1000x). For identification of the taxa, well-established cyanobacteria identifier books and floras were used. The phytoplankton count was performed with an inverted microscope Inverso (Medline Scientific, Chalgrove, Oxon, UK), equipped with a high definition digital camera using the Utermöhl method (Utermöhl 1958). For numerous species, at least 100 specimens were counted (Lund et al. 1958). The algal biovolume was calculated on the basis of formulas for geometric shapes (Hillebrand et al. 2002). The translation between biovolume and fresh biomass was performed according to Wetzel and Likens (2000). The full list for taxonomic composition and abundance of the phytoplankton species by stations is presented in Suppl. material 1.

### Determination of the ecological status by the phytoplankton indicator

The ecological status (ES) was determined by the HLPI method (Borics et al. 2018), intercalibrated for common intercalibration type L–EC1 (Lowland very shallow hard water) with the following characteristics: altitude < 200 m; depth < 6 m; conductivity 300-1000 μS cm^–1^; alkalinity 1-4 meq l^–1^ HCO_3_.

As a metric unit for taxonomic composition, the HLPI index contains the Q index (Padisák et al. 2006), based on the functional groups (FGs) of the phytoplankton. The FGs of the phytoplankton species were determined following Reynolds et al. (2002), Padisák et al. (2009) and Borics et al. (2015). Only those FGs, which have a relative biovolume > 5%, were identified.

### Analysis for presence of cyanotoxins by ELISA

Collected non-fixed water samples in September were analysed for the presence of the cyanotoxins microcystins/nodularins, cylindrospermopsin and saxitoxins by using the Microcystins/Nodularins (ADDA), Cylindrospermopsin and Saxitoxin (PSP) ELISA Kits (Abraxis LLC, Warminster, PA, USA). The microcystins/nodularins test is an indirect competitive ELISA for the congener-independent detection of microcystins and nodularins, based on the recognition of microcystins, nodularins and their congeners by specific antibodies. The cylindrospermopsin and saxitoxin tests are direct competitive ELISA’s for detection of cylindrospermopsin and saxitoxins. They are also based on the recognition of cylindrospermopsin or saxitoxins by specific antibodies.

All ELISA tests were performed in accordance with manufacturers' instructions. The intensity of the blue colour is inversely proportional to the concentration of microcystins, cylindrospermopsin or saxitoxins present in the sample. The coloured reactions were recorded by measuring the absorbance at 450 nm on a microplate reader ELx800™ (BioTek Instruments Inc., Winooski, VT, USA). The concentrations of the samples are determined by interpolation using the standard curve constructed with each run. The detection limit of the Microcystins/Nodularins ELISA kit is 0.15 ppb (μg.l^–1^). The mean lower detection limit of the cylindrospermopsin assay is about 0.05 ppb (μg.l^–1^). The detection limit of the Saxitoxin ELISA kit is 0.02 ppb (μg.l^–1^).

## Results

### Environmental conditions

Lake Vaya is a shallow, polymictic lake whose salinity varies slightly from 0.4 to 0.5‰ (Table 1). It is characterized by low transparency, which decreases from 0.5 m during the spring to 0.1 m at the end of the summer due to the growth intensity of the phytoplankton community. The proximity of the rivers that flow into the Lake determine the lower transparency of the Vaya-West station. The waters of the Lake are alkaline and, in July, the pH reaches 10.8. Over the entire study period, we found oxygen supersaturation of the surface layer up to > 200% during the summer months.

### Taxonomic composition and abundance of the phytoplankton

**Figure 2.**
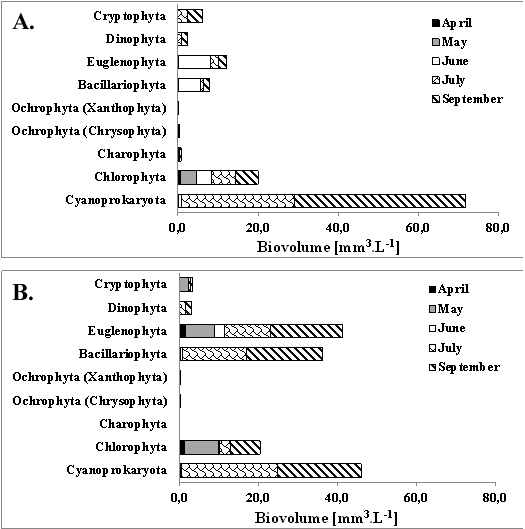
Seasonal succession in Lake Vaya according to the biovolume: **(A)** –Vaya-East, **(B)** – Vaya-West.

Analyzing the phytoplankton composition of Lake Vaya from April to September 2018, we identified 100 taxa belonging to eight phytoplankton groups (Cyanobacteria, Chlorophyta, Charophyta, Ochrophyta, Bacillariophyta, Euglenophyta, Dinophyta and Cryptophyta), amongst which Cyanobacteria were dominant during July and September (Fig. 2, Table 2).

**Figure 3.**
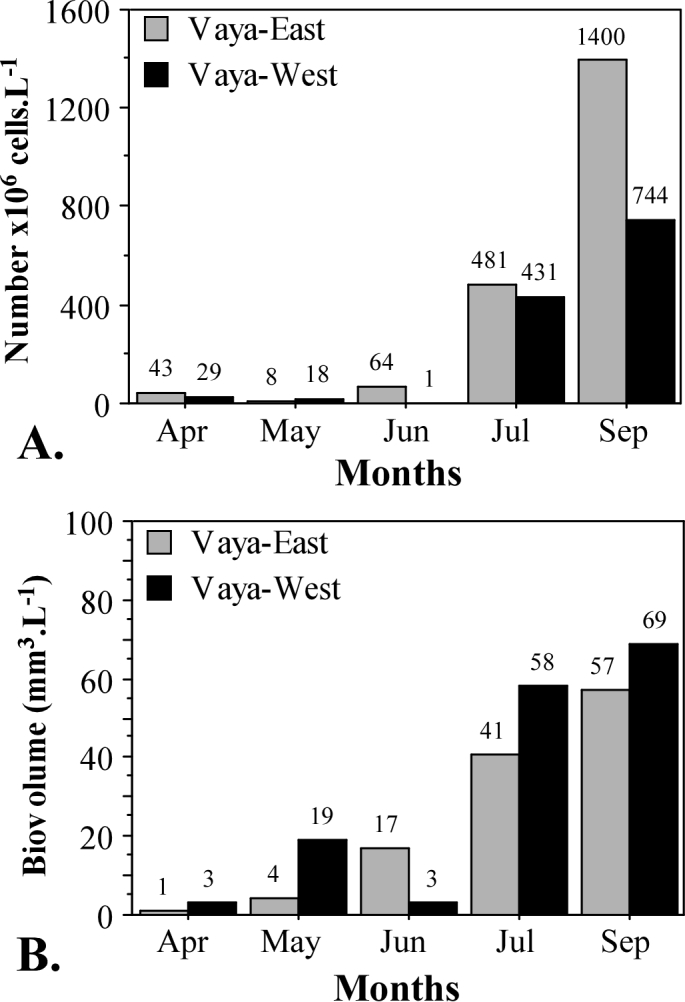
Total phytoplankton abundance **(A)** and total biovolume **(B)** during the study period.

In both parts of the Lake, the phytoplankton numbers/total biovolume increased steadily from spring (April, May) to autumn (September) (Fig. 3). From July to September, phytoplankton abundance tripled in the eastern part (up to 1400x10^6^ cells l^–1^) and doubled in the western part of the Lake (up to 744x10^6^ cells l^–1^) (Fig. 3A). A similar situation was observed for the overall biovolume (Fig. 3B). In both parts of the Lake, the July and September values were significantly higher than those from April to June (Fig. 3B). From April to September, the biovolume increased from 1 mm^3^ l^–1^ to 57 mm^3^ l^–1^ at the Vaya-East station and from 3 mm^3^ l^–1^ to 69 mm^3^ l^–1^ at the Vaya-West station.

During the study period, the two parts of the Lake (East and West) showed different qualitative and quantitative composition of the phytoplankton. In the eastern part, a higher phytoplankton density was observed. Ochrophytes, which dominated there in April, together with the cyanobacteria and chlorophytes, were not represented in the western part of the lake. The eastern part was richer in cyanobacterial species with a relative biovolume greater than 5% and there was a bloom of *Planktothrix agardhii* during the summer and autumn. The other dominant cyanobacterial species were representatives of the orders Nostocales (*Anabaenopsis elenkinii*, *Aphanizomenon klebanii* and *Dolichospermum flos-aquae*) and Synechococcales (*Snowella litoralis*), while dominant in the western part were representatives of the Synechococcales (*Limnothrix redekei*, *Phormidesmis molle*) and Oscillatoriales (*Planktothrix agardhii*, *Planktothrix isothrix*). The greater taxonomic diversity amongst the dominant diatoms (five species) and euglenophytes (eight species) in the western part is noticeable compared to the eastern part, where the two divisions were represented by two species each. Interestingly, the diatom species dominating in the eastern part in May and June (*Aulacoseira granulata, Cyclotella meneghiniana*) were much better represented in the western part in July and September, while their biovolume decreased in the eastern part. Chlorophytes (*Coelastrum microporum*, *Oocystis marssonii*, *Pandorina morum*) dominated in both parts of the Lake mainly in spring and cyanobacteria (*Planktothrix agardhii*) during summer and autumn (Table 2).

**Figure 4.**
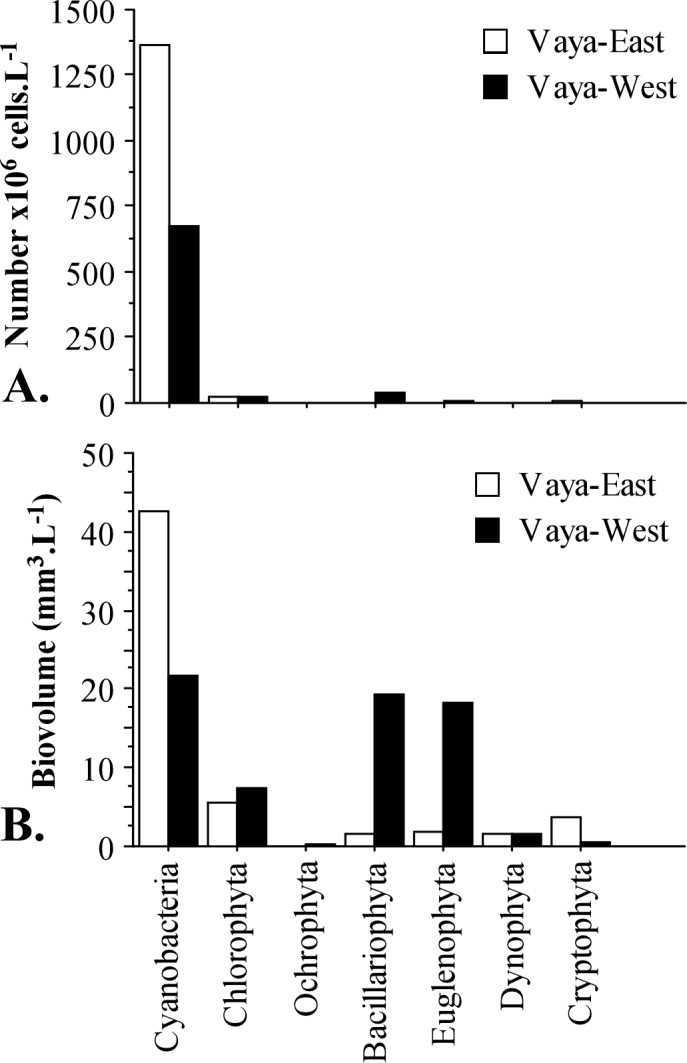
Phytoplankton abundance of the taxonomic groups **(A)** and biovolume of the taxonomic groups **(B)** in Lake Vaya during September.

**Figure 5.**
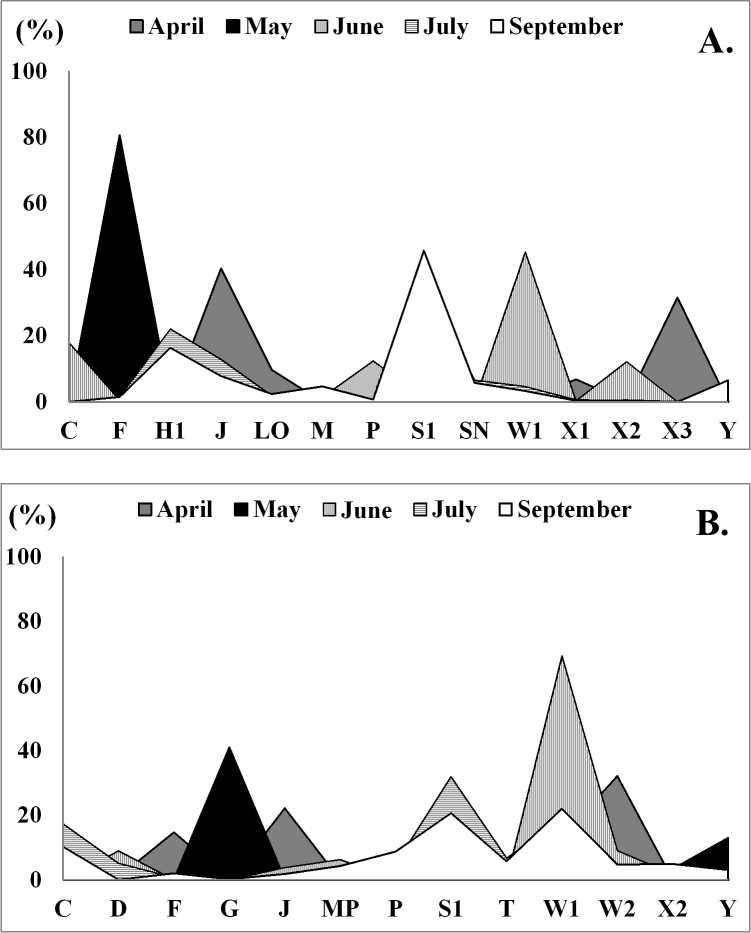
Seasonal succession of the relative biovolume of the main FGs (%) in Vaya-East **(A)** and Vaya-West **(B)**.

In June, euglenophytes (dominated by *Euglenaformis proxima*), diatoms (*Aulacoseira granulata* and *Cyclotella meneghiniana*) and chlorophytes (*Pseudopediastrum boryanum*) were most abundant in the eastern part of the Lake, while only euglenophytes and diatoms were present in the western part (Table 2, Fig. 4 and Fig. 5). In July, the dominance of euglenophytes and diatoms was replaced by that of cyanobacteria: *Planktothrix agardhii* in blooming concentration, *Anabaenopsis elenkinii*, *Aphanizomenon klebahnii* and *Dolichospermum flos-aquae* in Vaya-East; *Planktothrix agardhii*, *P. isothrix* and *Phormidesmis molle* in Vaya-West. This trend continued in September and was more pronounced in the eastern part of the Lake.

The cyanobacterial bloom and the detected microcystins and cylindrospermopsin in the water samples from September encouraged us to perform a detailed study of the phytoplankton during this month.

In September, cyanobacteria showed the highest numbers (1365.7x10^6^ cells l^–1^) in the eastern part of the Lake, followed by chlorophytes (24.7x10^6^ cells l^–1^), cryptophytes (4.41x10^6^ cells l^–1^) and diatoms (2.65x10^6^ cells l^–1^). Euglenophytes (0.69x10^6^ cells l^–1^) and ochrophytes (0.10x10^6^ cells ^–1^) were observed in relatively low numbers (Fig. 4A; Suppl. material 1). The total phytoplankton biovolume in this part of Lake Vaya was 57.5 mm^3^ l^–1^. Values of the biovolume followed the same trend as the numbers being the highest for cyanobacteria (42.7 mm^3^ l^–1^), followed by chlorophytes (5.59 mm^3^ l^–1^) and cryptophytes (3.76 mm^3^ l^–1^) (Fig. 4B; Suppl. material 1).

In Vaya-West, during September, cyanobacteria showed again the greatest numbers (674.6x10^6^ cells l^–1^). Unlike the eastern part, however, the second place was occupied by diatoms (35.79x10^6^ cells l^–1^), which displaced chlorophytes (24.26x10^6^ cells l^–1^) to third place. The following taxonomic groups were euglenophytes (5.7x10^6^ cells l^–1^), ochrophytes (0.76x10^6^ cells l^–1^), dinophytes (0.32x10^6^ cells l^–1^) and cryptophytes (0.26x10^6^ cells l^–1^) (Fig. 4B; Suppl. material 1). The total phytoplankton biovolume in the western part of Vaya-Lake in September was 68.6 mm^3^ l^–1^ dominated again by cyanobacteria (21.19 mm^3^ l^–1^), followed by diatoms (19.18 mm^3^ l^–1^) and euglenophytes (18.30 mm^3^ l^–1^).

### Ecological status (ES) of Lake Vaya

The application of the HPLI method showed that both stations of the Lake (Vaya-East and Vaya-West) are eutrophied (Table 3). The ecological status of Vaya-East is moderate, while the Vaya-West ecological status is of lower quality class – poor. The bad ecological status of Vaya-West is due to the significantly higher mean value of chlorophyll-*a*, as the values of the taxonomic index (Q index or normalized Ecological Quality Ratio /EQR/ of the Q index) at both stations do not differ significantly.

Effects of the eutrophication, which immediately affect the primary producers, were present at both (Vaya-East and Vaya-West) points – intensive, prolonged blooming of eutrophic cyanobacteria (Fig. 4). There is evidence that the dominant cyanobacterial species in blooming concentrations produce cyanotoxins. The physicochemical parameters confirmed the bad quality of the water – alkaline pH, low transparency and oxygen supersaturation of the surface layers during the day (Table 1).

### Seasonal succession of the main functional groups (FGs) of the phytoplankton

During the study period, 14 main FGs were identified in Vaya-East and 13 FGs in Vaya-West (Fig. 5). Eight of them were common to both stations: **C, F, J, P, S1, W1, X2** and **Y**. Except for functional group **F**, the others are typical inhabitants of nutrient-rich conditions in turbid eutrophic lakes (Padisák et al. 2009).

Although located in the same Lake, the two stations had different seasonal succession of the main FGs, which reflects the specifics of the two habitats. At Vaya-West station, there is an increased growth of macrophytes (*Potamogeton pectinatus*, *Lemna minor*) and the seasonal succession takes place in the following sequence: **W2→W1,G→W1→S1,W1→W1,S1** (Fig. 5). Dominants or subdomains throughout the growing season were Euglenophyta from FGs **W1** and **W2**, with their strongest development in June (70% relative biovolume). Other flagellated species from FGs **G** (green algae) and **Y** (cryptophytes) were co-dominants in May. After the peak of Euglenophyta in June, the presence of FGs tolerant to reduced light conditions increased in the middle and late summer. This is FG **S1** (*Planktothrix spp*.) that is attached to persistently mixed layers, in which light is increasingly the limiting constraint (Padisák et al. 2009). The presence of **Tc** (*Phormidesmis*) and diatoms from **MP** (*Navicula recens*) is explained by the abundance of emergent macropytes (Table 2). The lack of stratification and continuous mixing are favourable for the growth of heavy diatoms from **C** (*Cyclotella meneghiniana*) and **P** (*Aulacoseira granulata*).

The seasonal succession at Vaya-East, located in the open Lake, was dominated by other functional groups: **J→F→W1→S1,H1** (Fig. 5). In April, the phytoplankton was dominated by FG **J**. According to Borics et al. (2012), the most characteristic elements of the phytoplankton in well-mixed open lakes are the chlorococcalean green algae (**J**). For a short time, FG **F** (*Oocystis marssonii* - 80.7%) was found as a monodominant in May, although, according to literature, FG **F** is typical for clear, deeply-mixed meso-eutrophic lakes (Reynolds et al. 2002). In June, the number of main FGs increases and the biovolume is distributed more evenly between diatoms (**C, P**) and flagellated species (**X2, W1**). From July to September, intensive blooms of eutrophic cyanobacteria **S1** (*Planktothrix agardhii*) with subdomains **H1** (*Anabaenopsis elenkinii, Aphanizomenon klebahnii, Dolichospermumflos-aquae*) and **S_N_** (*Raphidiopsis raciborskii*) appeared and remained (Fig. 5, Suppl. material 1). A probable reason for the dominance of FGs **S1, H1** and **S_N_** is that they are strong light competitors (Reynolds 2006). The situation, in which, during the later succession state, the lakes are dominated by several species or a certain functional group of algae, is known as equilibrium states or steady-state assemblages (Naselli-Flores et al. 2003).

### Taxonomic composition of Cyanobacteria, blooms and cyanotoxins

In the present study, 22 cyanobacterial species belonging to 18 genera have been identified. Many of the identified cyanobacteria were previously reported as producers of cyanotoxins – *Dolichospermum flos-aquae*, *Microcystis aeruginosa*, *Microcystis wesenbergii*, *Planktothrix agardhii* and *Raphidiopsis raciborskii*. Previous studies on the phytoplankton composition of Lake Vaya registered cyanobacterial blooms with dominance of *Microcystis wesenbergii*, *Aphanizomenon gracile*, *Aphanizomenon flos-aquae* and *Dolichospermum spiroides* (Stoyneva 2003).

During September 2018, a proven producer of cyanotoxins, *Planktothrix agardhii*, was found in blooming concentration in the eastern part of the Lake. Its biomass was 24.89 mg.l^–1^, which exceeded twice the accepted WHO moderate risk threshold of 10 mg.l^–1^. Other bloom-forming species, such as *Anabaenopsis elenkinii* (3.5 mg.l^–1^), *Aphanizomenon klebahnii* (3.09 mg.l^–1^), *Dolichospermum flos-aquae* (2.2 mg.l^–1^) *Microcystis aeruginosa* (1.65 mg.l^–1^), *Raphidiopsis raciborskii* (1.66 mg.l^–1^) and *Microcystis wesenbergii* (1 mg.l^–1^), were well-represented in a cyanobacterial bloom although their biomass was below the level of the bloom concentrations.

*Planktothrix agardhii* (9.3 mg.l^-1^) was found also in the western part of the Lake at a concentration close to a moderate risk threshold of 10 mg.l^-1^. Other cyanobacterial species represented in this part of the Lake were *Anabaenopsis elenkinii* (0.7 mg.l^-1^), *Aphanizomenon klebahnii* (0.1 mg.l^-1^), *Dolichospermum flos-aquae* (0.6 mg.l^-1^) *Microcystis aeruginosa* (1.05 mg.l^-1^), *Phormidesmis molle* (3.9 mg.l^-1^) and *Planktothrix isothrix* (4.7 mg.l^-1^). *Microcystis wesenbergii* was not detected in this part of the lake.

**Figure 6.**
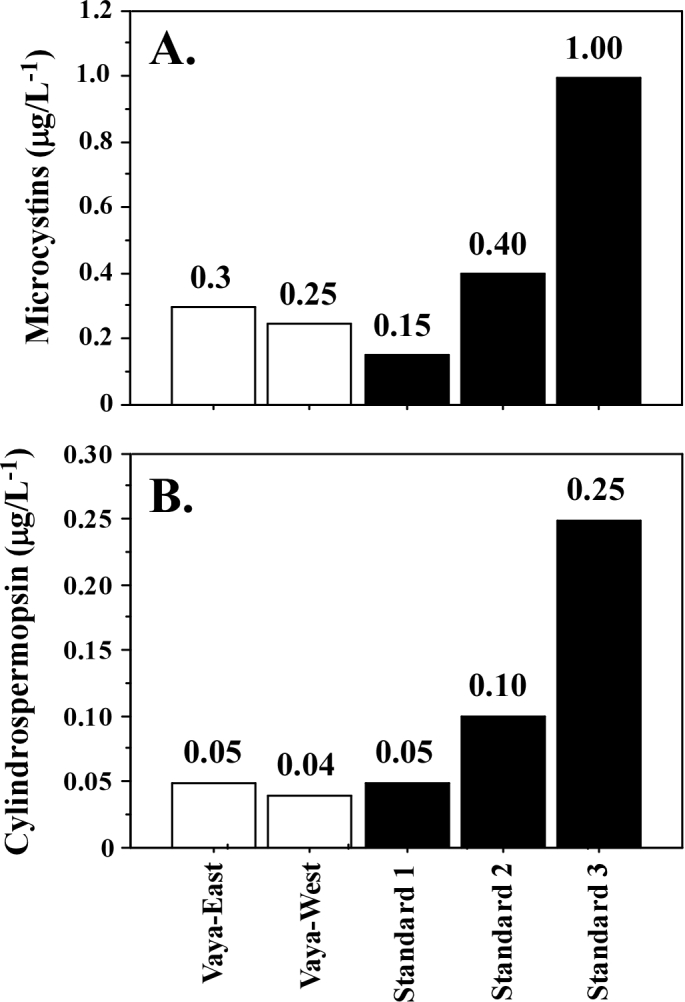
Presence of microcystins **(A)** and cylindrospermopsin **(B)** in the water samples from September and tested by ELISA.

Our ELISA tests for cyanotoxins in September, showed 0.3 μg.l^–1^ and 0.25 μg.l^–1^ microcystins and 0.05 μg.l^–1^ and 0.04 μg.l^–1^ cylindrospermopsin in the eastern and western part, respectively. (Fig. 6). Saxitoxins were not detected. Previous analyzes for cyanotoxins from 2005 and 2011-2013 conducted by HPLC, did not detect the presence of cyanotoxins (Pavlova et al. 2014; Pavlova et al. 2015; Stoyneva-Gärtner et al. 2017).

We have reported for the first time cylindrospermopsin from a Bulgarian water basin (Teneva et al. 2019).

The cyanotoxins detected in this study, albeit in quantities below the maximum levels, are a warning sign, even more so as Lake Vaya is located on the Via Pontica migratory road and is one of the most important stations for bird migration on the Bulgarian Black Sea coast.

## Discussion

Our study presents data on the taxonomic composition and quantitative parameters of the phytoplankton in Lake Vaya for the period April-September 2018, with emphasis on Cyanobacteria and their toxins. Taking into account the conservation status of Lake Vaya, on the one hand and the ecological threat of the cyanotoxins on the other, we believe that this study is a contribution to the necessary status assessment of the Lake.

Observed physico-chemical parameters are in line with the conclusions of the last annual evaluation report on the current condition of the Black Sea region waters (Black Sea Basin Directorate - Varna 2015) where it was specifically noted that Lake Vaya "has a bad chemical and ecological status". According to the latest hydrochemical studies of the waters of Lake Vaya, conducted in March 2017, it was concluded that there are negative trends in the hydrochemical development of Lake Vaya (Agafonova 2018).

Analyzing the taxonomic composition of the phytoplankton, we found that, in both parts of the Lake, the phytoplankton numbers/total biovolume increased steadily from April to September. The eastern and western part of the Lake showed different qualitative and quantitative structure of the phytoplankton. Comparing our data with those reported for the period 2004-2006 by Dimitrova et al. (2014a), Dimitrova et al. (2014b), a slight increase in the number of Cyanobacteria and almost 50% reduction of Chlorophyta (24x10^6^ cells l^–1^ for 2018 and 42.6x10^6^ cells l^–1^ for 2004-2006) can be seen.

We have determined the ES of the lake by applying the Hungarian Lake Phytoplankton Index (HLPI) method. The method is sensitive to the eutrophication pressure (total phosphorus and total nitrogen), as well as to the impairment of the balance between primary producers – macrophytes and algae (Borics et al. 2018). According to our data, the ecological status of Vaya-East is moderate, while for the Vaya-West a lower quality class (poor) was determined.

Anthropogenic eutrophication is defined as enriching water with nutrients that cause accelerated growth of algae and higher plants and this leads to undesirable disturbance of organisms’ balance and water quality (Common implementation strategy for the water framework directive (2000/60/EC) 2009). It is known that significant undesirable disturbance of the aquatic ecosystem, resulting from the accelerated development of phytoplankton, is enhanced from “Moderate” to “Bad” ecological status. This includes worsening conditions for benthic invertebrates and fish as a result of increased sedimentation of organic matter, oxygen deficiency during the night, release of hydrogen sulphide and changes in habitat availability. Eutrophic waters, assessed in Moderate/Poor ecological status, compromise the achievement of the objectives for Natura 2000.

Identified functional groups (FGs) of the phytoplankton in Vaya-West support the opinion of Borics et al. (2012) that not always the dominance of macrophytes in shallow eutrophic lakes leads to clear water state, as dense algae populations may grow in small lakes amongst macrophytes. At Vaya-West, we observed a continuous intense phytoplankton growth in the middle and late summer, regardless of the abundance of macrophytes. The analysis of the diversity of the main FGs confirmed that the most competitive FGs of the mobile species are euglenophytes (**W1, W2**), cryptophytes (**Y**) and motile green algae (**G**). They are able to actively change their position in the water column by avoiding shading of the macrophytes and the pressure of the zooplankton.

We believe that the lack of cyanobacterial blooms and lower levels of cyanotoxins in the western part of the lake is associated with the increased presence of macrophytes. During the sampling in September, the presence of *Potamogeton pectinatus* (coverage 30%, abundance 20%) and *Lemna minor* was observed. This confirms what has been reported so far in literature that macrophytes do not occur massively in the presence of blooms. In addition, the concentration of cyanotoxins (MCs, CYL) detected in such samples is lower. In the eastern part, where a bloom of *Planktothrix agardhii* was observed, macrophytes were scarce and a higher concentration of cyanotoxins (MCs, CYL) was detected in the water sample. These data allow us to speculate that the cyanotoxins released during the cyanobacterial bloom inhibit the growth of macrophytes. During cyanobacterial blooms in eutrophic waters, many macrophytes disappear (Mohamed 2017). Other authors argue that macrophytes inhibit the growth and development of cyanobacteria (Shan et al. 2015). These relationships are not yet well understood and currently are the subject of intensive research.

## Conclusions

The analysis showed a tendency of increase in phytoplankton abundance (numbers, biovolume) and changes in its structure in comparison with previous data from the last 30 years. Cyanobacteria were imposed as the dominant group, displacing Chlorophyta and Bacillariophyta. The application of the HLPI method to assess the ecological status of Lake Vaya in 2018 showed that the Lake was eutrophied with the ecological status from moderate (Vaya-East) to poor (Vaya-West). The two stations showed different seasonal succession of the main FGs. The abundance of macrophytes (*Potamogeton pectinatus* and *Lemna minor*) in the western part influences the functional structure of the phytoplankton community and facilitates the dominance of flagellated species from FGs **W1, W2, Y** and **G**. In the open eastern part of the Lake, the filamentous eutrophic cyanobacteria (FGs: **S1, SN** and **H1**), as well as green chlorococcal algae from **J**, were best suited.

Cyanobacteria were present in the Lake throughout the study period, but became a dominant group in September, building cyanobacterial "blooms" of toxin-producing species. The formation of "blooms", the presence of toxin-producing species, as well as the detected cyanotoxins (microcystins and cylindrospermopsin) in September, are a warning sign that indicates the need for continued monitoring of cyanobacterial composition, blooming and presence of cyanotoxins in Lake Vaya.

Cyanobacterial blooms and the toxins released by them pose a risk to both humans and the ecosystems including nesting and migratory birds. Lake Vaya is actively used for fishing and toxins accumulated in fish can be transmitted along the food chain to human consumers. The problem with cyanobacterial blooms and cyanotoxins is not only local. Taking into account the onset of climate change, this problem applies to almost every water basin in the world. Information on the spread and occurrence of cyanobacterial blooms and associated cyanotoxins is important in order to take adequate actions to limit and/or eliminate them.

## Supplementary Material

26907B39-2006-5F39-8EE5-FC6DD61B0F01 10.3897/BDJ.8.e57507.suppl1 Supplementary material 1 List of the phytoplankton species found in Lake Vaya Data type Taxonomical composition of the phytoplankton in Lake Vaya. Brief description Phytoplankton composition with an emphasis of Cyanobacteria and their toxins as an indicator for the ecological status of Lake Vaya (Bulgaria) – part of the Via Pontica migration route. File: oo_476945.doc https://binary.pensoft.net/file/476945 Ivanka Teneva, Detelina Belkinova, Rumen Mladenov, Plamen Stoyanov, Dzhemal Moten, Diyana Basheva, Stefan Kazakov, Balik Dzhambazov

## Figures and Tables

**Table 1.** Morphometric, physical and chemical parameters. T - Temperature; DO - Dissolved oxygen; OS - Oxygen saturation; Cond - Conductivity; n.a. - not applied

**Morphometric parameters**										
**Altitude [m a.s.l.**]					0 m					
**Max. length**					9.6 km					
**Max. width**					5 km					
**Area [ha**]					27.60 km^2^					
**Maximum depth [m**]					2.5 m					
**Physical and chemical parameters**										
	**April**		**May**		**June**		**July**		**September**	
**Sample station**	**Vaya-East**	**Vaya-West**	**Vaya-East**	**Vaya-West**	**Vaya-East**	**Vaya-West**	**Vaya-East**	**Vaya-West**	**Vaya-East**	**Vaya-West**
**T [^o^ C**]	15.7	16.4	22.8	22.5	26.7	24.5	27.7	28.5	24.5	24.9
**DO [mg.lL^-1^**]	9.1	10.68	5.735	10.15	12.37	5.23	16.5	16.8	9.14	19.45
**OS** [%]	9.1	108	66.5	115	154	70	>200	>200	108.6	>200
**рН**	8.85	8.48	8.56	8.61	8.89	7.8	10.74	10.84	8.31	8.32
**Cond [μS.cm^-1^**]	1048	862	548.5	858	932	1043	880	921	1016	1136
**Salinity** [‰]	0.5	0.4	0.4	0.4	0.4	0.5	0.4	0.4	0.4	0.5
**Transparency [m**]	0.5	0.4	1.3	0.4	0.45	0.25	0.3	0.2	0.1	0.1
**Chlorophyll-a, [μg.l^-1^**]	71.08	61.21	10.86	190.39	93.55	29.62	201.39	331.71	n.a.	n.a

**Table 2.** Dominant species (with relative biovolume >5%) and their functional groups (FGs) in Lake Vaya.

**Phytoplankton** **(dominant species)**	**Relative biovolume (%) during the months**	**FGs**	**Phytoplankton** **(dominant species)**	**Relative biovolume (%) during the months**	**FGs**
**Vaya-East**	A/M/J/J/S		**Vaya-West**	A/M/J/J/S	
** Cyanobacteria **			** Cyanobacteria **		
*Anabaenopsis elenkinii*	0/0/0/8/6	**H1**	*Limnothrix redekei*	15/0/0/0/0	**S1**
*Aphanizomenon klebahnii*	0/0/0/8/5	**H1**	*Phormidesmis molle*	0/0/0/7/6	**T_C_**
*Dolichospermum flos-aquae*	0/0/0/5/4	**H1**	*Planktothrix agardhii*	0/0/0/23/14	**S1**
*Planktothrix agardhii*	0/0/0/33/43	**S1**	*Planktothrix isothrix*	0/0/0/9/7	**S1**
*Snowella litoralis*	9/0/0/0/0	**L_0_**			
** Chlorophyta **			** Chlorophyta **		
*Chlamydomonas* sp.	0/0/12/0/0	**X2**	*Coelastrum microporum*	21/0/0/0/1	**J**
*Coelastrum microporum*	29/0/0/0/0	**J**	*Dictyosphaerium* sp.	10/0/0/0/0	**F**
*Oocystis marssonii*	0/81/0/0/0	**F**	*Eudorina elegans*	0/5/0/0/0	**G**
*Pectinodesmus pectinatus*	5/0/0/0/0	**J**	*Oocystis marssonii*	5/0/0/1/2	**F**
*Pseudopediastrum boryanum*	0/1/2/7/3	**J**	*Pandorina morum*	0/36/0/0/0	**G**
** Charophyta **					
*Closterium acutum*	5/0/0/0/0	**P**			
** Ochrophyta **					
Chrysophyta cysts	31/0/0/0/0	**X3**			
** Bacillariophyta **			** Bacillariophyta **		
*Aulacoseira granulata*	0/2/12/0/0	**P**	*Aulacoseira granulata*	0/0/0/5/9	**P**
*Cyclotella meneghiniana*	0/0/18/0/0	**C**	*Cyclotella meneghiniana*	0/0/0/17/10	**C**
			*Fragilaria acus*	0/0/9/0/0	**D**
			*Navicula recens*	0/0/6/0/0	**MP**
			*Stephanodiscus minutulus*	1/0/0/5/5	**D**
** Euglenophyta **			** Euglenophyta **		
*Euglena variabilis*	0/0/6/0/0	**W1**	*Euglena chlamydophora*	13/0/0/0/0	**W1**
*Euglenaformis proxima*	0/5/37/0/0	**W1**	*Euglenaformis proxima*	0/29/26/3/5	**W1**
			*Euglena variabilis*	0/8/21/1/1	**W1**
			*Euglena* sp.	0/0/17/0/0	**W1**
			*Lepocinclis oxyuris*	0/0/0/0/7	**W1**
			*Lepocinclis* sp.	0/1/0/9/4	**W1**
			*Strombomonas* sp.	0/2/9/1/3	**W2**
			*Trachelomonas* sp.	32/0/0/0	**W2**
** Cryptophyta **			** Cryptophyta **		
*Cryptomonas erosa*	0/0/0/6/6	**Y**	*Cryptomonas erosa*	0/13/0/1/1	**Y**

**Table 3.** Ecological status of Lake Vaya. HLPI: Hungarian Lake Phytoplankton Index, Chl-*a*: chlorophyll-*a* (µg.l^–1^), EQRChl-*a*: normalized EQR of the Chlorophyll-*a* metric, Q index: composition metric, based on functional groups, EQR_Q_: normalized EQR of the Q index.

	**Chl-*a* (µg.l^-1^**)	**EQR _Chl -*а*_**	**Q**	**EQR_Q_**	**HLPI**	**Ecological status**
**Vaya-West**	153.23	0.24	4.64	0.66	0.38	poor
**Vaya-East**	94.22	0.37	5.36	0.69	0.47	moderate
