# Supplementary material for: Phytoplankton composition with an emphasis of Cyanobacteria and their toxins as an indicator for the ecological status of Lake Vaya (Bulgaria) – part of the Via Pontica migration route
Source: Biodivers Data J. 2020 Dec 16;8:e57507. doi: 10.3897/BDJ.8.e57507 (PMC7758307; doi:10.3897/BDJ.8.e57507)
Supplement: Supplementary material 1 — List of the phytoplankton species found in Lake Vaya [file bdj-08-e57507-s001.doc]

**Appendix 1.** Taxonomical composition of the phytoplankton in Vaya Lake.

| **Taxon – Vaya-East** | **Numbers**  **[x10^6^ cells.L^-1^]** | **Biovolume [mg.L^-1^]** | **Relative biovolume [%]** |
| --- | --- | --- | --- |
| **Cyanobacteria** | **1365.72** | **42.71** | **74.30** |
| *Anabaenopsis elenkinii* V.V.Miller 1923 | 39.23 | 3.61 | 6.28 |
| *Aphanizomenon klebahnii* Elenkin ex Pechar 2008 | 93.65 | 3.09 | 5.38 |
| *Aphanocapsa elachista* West & G.S.West 1894 | 1.61 | 0.00 | 0.01 |
| *Aphanocapsa incerta* (Lemmermann) G.Cronberg & Komárek 1994 | 3.43 | 0.00 | 0.00 |
| *Dolichospermum flos-aquae* (Brébisson ex Bornet & Flahault) P.Wacklin et al. 2009 | 34.32 | 2.23 | 3.88 |
| *Limnococcus limneticus* (Lemmermann) Komárková et al. 2010 | 0.40 | 0.07 | 0.13 |
| *Limnothrix planctonica* (Woloszynska) Meffert 1988 | 49.52 | 0.15 | 0.27 |
| *Merismopedia tenuissima* Lemmermann 1898 | 3.23 | 0.00 | 0.00 |
| *Microcystis aeruginosa* (Kützing) Kützing 1846 | 25.53 | 1.66 | 2.89 |
| *Microcystis wesenbergii* (Komárek) Komárek ex Komárek in Joosen 2006 | 9.08 | 1.03 | 1.79 |
| *Myxobaktron sp*. | 2.45 | 0.03 | 0.05 |
| *Planktolyngbya limnetica* (Lemmermann) Komárková-Legnerová & Cronberg 1992 | 76.49 | 0.24 | 0.41 |
| *Planktothrix agardhii* (Gomont) Anagnostidis & Komárek 1988 | 888.95 | 24.89 | 43.30 |
| *Planktothrix isothrix* (Skuja) Komárek & Komárková 2004 | 35.30 | 1.02 | 1.78 |
| *Pseudanabaena catenata* Lauterborn 1915 | 17.16 | 0.32 | 0.56 |
| *Raphidiopsis mediterranea* Skuja 1937 | 43.15 | 1.64 | 2.85 |
| *Raphidiopsis raciborskii* (Woloszynska) Aguilera et al. 2018 | 14.71 | 1.66 | 2.89 |
| *Snowella litoralis* (Häyrén) Komárek & Hindák 1988 | 8.12 | 0.11 | 0.20 |
| *Sphaerospermopsis aphanizomenoides* (Forti) Zapomelová et al. 2010 | 5.15 | 0.47 | 0.82 |
| *Synechocystis salina* Wislouch 1924 | 14.22 | 0.47 | 0.82 |
| **Chlorophyta** | **24.86** | **5.59** | **9.73** |
| *Coelastrum pseudomicroporum* Korshikov 1953 | 0.40 | 0.05 | 0.09 |
| *Desmodesmus communis* (E.Hegewald) E.Hegewald 2000 | 0.20 | 0.04 | 0.07 |
| *Desmodesmus protuberans* (F.E.Fritsch & M.F.Rich) E.Hegewald 2000 | 0.40 | 0.11 | 0.19 |
| *Desmodesmus subspicatus* (Chodat) E.Hegewald & A.Schmidt in E.Hegewald 2000 | 1.96 | 0.03 | 0.05 |
| *Dictyosphaerium sp*. | 2.07 | 0.13 | 0.23 |
| *Golenkinia radiata* Chodat 1894 | 3.43 | 1.80 | 3.12 |
| *Lagerheimia subsalsa* Lemmermann 1898 | 1.47 | 0.34 | 0.59 |
| *Micractinium crassisetum* Hortobágyi 1973 | 4.43 | 0.50 | 0.87 |
| *Monoraphidium arcuatum* (Korshikov) Hindák 1970 | 0.15 | 0.02 | 0.03 |
| *Monoraphidium contortum* (Thuret) Komárková-Legnerová in Fott 1969 | 0.10 | 0.00 | 0.00 |
| *Monoraphidium griffithii* (Berkeley) Komárková-Legnerová 1969 | 0.10 | 0.01 | 0.02 |
| *Monoraphidium minutum* (Nägeli) Komárková-Legnerová 1969 | 0.10 | 0.00 | 0.01 |
| *Nephrochlamys subsolitaria* (G.S.West) Korshikov 1953 | 1.47 | 0.13 | 0.23 |
| *Oocystis lacustris* Chodat 1897 | 0.20 | 0.05 | 0.09 |
| *Oocystis marssonii* Lemmermann 1898 | 0.10 | 0.13 | 0.22 |
| *Pectinodesmus pectinatus* (Meyen) E.Hegewald, M.Wolf, Al.Keller, Friedl & Krienitz 2010 | 0.40 | 0.05 | 0.09 |
| *Phacotus lenticularis* (Ehrenberg) Diesing 1866 | 0.10 | 0.09 | 0.16 |
| *Pseudopediastrum boryanum* (Turpin) E.Hegewald in Buchheim et al. 2005 | 0.05 | 1.50 | 2.61 |
| *Pseudoschroederia robusta* (Korshikov) E.Hegewald & E.Schnepf 1986 | 0.10 | 0.04 | 0.06 |
| *Schroederia spiralis* (Printz) Korshikov 1953 | 0.98 | 0.13 | 0.23 |
| *Tetradesmus lagerheimii* M.J.Wynne & Guiry 2016 | 2.94 | 0.04 | 0.08 |
| *Tetraëdron minimum* (A.Braun) Hansgirg 1888 | 0.98 | 0.15 | 0.26 |
| *Tetraëdron triangulare* Korshikov 1953 | 0.98 | 0.10 | 0.17 |
| *Tetrastrum staurogeniaeforme* (Schröder) Lemmermann 1900 | 1.61 | 0.10 | 0.18 |
| *Treubaria triappendiculata* C.Bernard 1908 | 0.10 | 0.04 | 0.07 |
| **Charophyta** | **0.35** | **0.44** | **0.76** |
| *Cosmarium sp*. | 0.10 | 0.03 | 0.06 |
| *Staurastrum chaetoceras* (Schröder) G.M.Smith 1924 | 0.15 | 0.30 | 0.53 |
| *Staurastrum gracile* Ralfs ex Ralfs 1848 | 0.10 | 0.10 | 0.18 |
| **Ochrophyta** | **0.10** | **0.05** | **0.09** |
| *Pseudostaurastrum limneticum* (Borge) Couté & Rousselin 1975 | 0.10 | 0.05 | 0.09 |
| **Bacillariophyta** | **2.65** | **1.55** | **2.69** |
| *Navicula sp*. | 0.49 | 0.06 | 0.10 |
| *Nitzschia acicularis* (Kützing) W.Smith 1853 | 0.98 | 0.10 | 0.18 |
| *Nitzschia dissipata* (Kützing) Rabenhorst 1860 | 0.05 | 0.01 | 0.02 |
| *Nitzschia palea* (Kützing) W.Smith 1856 | 0.15 | 0.07 | 0.13 |
| *Stephanodiscus hantzschii* Grunow in Cleve & Grunow 1880 | 0.98 | 1.30 | 2.26 |
| **Euglenophyta** | **0.69** | **1.94** | **3.37** |
| *Cryptoglena skujae* Marin & Melkonian in Marin et al. 2003 | 0.15 | 0.04 | 0.07 |
| *Euglena geniculata* Dujardin 1841 | 0.49 | 1.73 | 3.01 |
| *Monomorphina pyrum* (Ehrenberg) Mereschkowsky 1877 | 0.05 | 0.17 | 0.29 |
| **Dinophyta** | **1.03** | **1.45** | **2.52** |
| *Gymnodinium sp*. | 0.05 | 0.27 | 0.48 |
| *Katodinium sp*. | 0.98 | 1.18 | 2.05 |
| **Cryptophyta** | **4.41** | **3.76** | **6.54** |
| *Cryptomonas erosa* Ehrenberg 1832 | 1.47 | 3.48 | 6.06 |
| *Komma caudata* (L.Geitler) D.R.A.Hill 1991 | 2.94 | 0.28 | 0.48 |
| **Total:** | **1399.82** | **57.48** | **100.00** |
| **Taxon – Vaya-West** | **Numbers**  **[x10^6^ cells.L^-1^]** | **Biovolume [mg.L^-1^]** | **Relative biovolume [%]** |
| **Cyanobacteria** | **674.68** | **21.19** | **30.86** |
| *Anabaenopsis elenkinii* V.V.Miller 1923 | 7.73 | 0.71 | 1.04 |
| *Aphanizomenon klebahnii* Elenkin ex Pechar 2008 | 3.04 | 0.10 | 0.15 |
| *Aphanocapsa elachista* West & G.S.West 1894 | 2.60 | 0.00 | 0.01 |
| *Dolichospermum flos-aquae* (Brébisson ex Bornet & Flahault) P.Wacklin et al. 2009 | 9.29 | 0.60 | 0.88 |
| *Dolichospermum planctonicum* (Brunnthaler) Wacklin, L.Hoffmann & Komárek 2009 | 2.48 | 0.17 | 0.24 |
| *Glaucospira laxissima* (G.S.West) Simic, Komárek & Dordevic 2014 | 0.55 | 0.08 | 0.11 |
| *Limnococcus limneticus* (Lemmermann) Komárková et al. 2010 | 0.51 | 0.09 | 0.13 |
| *Limnothrix planctonica* (Woloszynska) Meffert 1988 | 33.68 | 0.10 | 0.15 |
| *Microcystis aeruginosa* (Kützing) Kützing 1846 | 16.19 | 1.05 | 1.53 |
| *Myxobaktron sp*. | 1.38 | 0.02 | 0.03 |
| *Phormidesmis molle* (Gomont) Turicchia, Ventura, Komárková & Komárek | 66.54 | 3.93 | 5.72 |
| *Planktothrix agardhii* (Gomont) Anagnostidis & Komárek 1988 | 331.32 | 9.28 | 13.51 |
| *Planktothrix isothrix* (Skuja) Komárek & Komárková 2004 | 162.90 | 4.72 | 6.88 |
| *Pseudanabaena catenata* Lauterborn 1915 | 13.53 | 0.22 | 0.32 |
| *Pseudanabaena sp*. | 21.54 | 0.07 | 0.10 |
| *Raphidiopsis mediterranea* Skuja 1937 | 1.38 | 0.05 | 0.08 |
| **Chlorophyta** | **24.26** | **7.43** | **10.82** |
| *Carteria multifilis* (Fresenius) O.Dill 1895 | 1.10 | 2.37 | 3.45 |
| *Carteria pseudoglobosa* Ettl 1979 | 1.10 | 0.77 | 1.12 |
| *Chlamydomonas reinhardtii* P.A.Dangeard 1888 | 1.10 | 0.23 | 0.33 |
| *Coelastrum microporum* Nägeli in A.Braun 1855 | 1.36 | 0.37 | 0.53 |
| *Coelastrum pseudomicroporum* Korshikov 1953 | 1.36 | 0.18 | 0.26 |
| *Desmodesmus communis* (E.Hegewald) E.Hegewald 2000 | 0.34 | 0.07 | 0.10 |
| *Desmodesmus intermedius* (Chodat) E.Hegewald 2000 | 3.31 | 0.13 | 0.18 |
| *Desmodesmus opoliensis* var. *mononensis* (Chodat) E.Hegewald 2000 | 1.10 | 0.07 | 0.11 |
| *Desmodesmus protuberans* (F.E.Fritsch & M.F.Rich) E.Hegewald 2000 | 0.34 | 0.06 | 0.08 |
| *Dictyosphaerium sp*. | 1.02 | 0.05 | 0.07 |
| *Elakatothrix gelatinosa* Wille 1898 | 0.13 | 0.00 | 0.01 |
| *Gonium pectorale* O.F.Müller 1773 | 0.68 | 0.06 | 0.09 |
| *Micractinium pusillum* Fresenius 1858 | 0.21 | 0.01 | 0.01 |
| *Monoraphidium arcuatum* (Korshikov) Hindák 1970 | 0.13 | 0.01 | 0.02 |
| *Monoraphidium circinale* (Nygaard) Nygaard 1979 | 0.83 | 0.06 | 0.09 |
| *Monoraphidium contortum* (Thuret) Komárková-Legnerová in Fott 1969 | 0.21 | 0.00 | 0.01 |
| *Monoraphidium griffithii* (Berkeley) Komárková-Legnerová 1969 | 0.09 | 0.01 | 0.02 |
| *Monoraphidium minutum* (Nägeli) Komárková-Legnerová 1969 | 2.21 | 0.14 | 0.21 |
| *Nephrochlamys subsolitaria* (G.S.West) Korshikov 1953 | 0.26 | 0.01 | 0.01 |
| *Oocystis lacustris* Chodat 1897 | 1.10 | 0.28 | 0.41 |
| *Oocystis marssonii* Lemmermann 1898 | 0.83 | 1.04 | 1.51 |
| *Pandorina morum* (O.F.Müller) Bory in J.V.Lamouroux et al. 1827 | 0.51 | 0.14 | 0.20 |
| *Phacotus lenticularis* (Ehrenberg) Diesing 1866 | 0.83 | 0.77 | 1.12 |
| *Pseudoschroederia robusta* (Korshikov) E.Hegewald & E.Schnepf 1986 | 0.21 | 0.07 | 0.10 |
| *Schroederia spiralis* (Printz) Korshikov 1953 | 1.66 | 0.38 | 0.55 |
| *Tetrastrum staurogeniaeforme* (Schröder) Lemmermann 1900 | 2.21 | 0.14 | 0.21 |
| **Ochrophyta** | **0.76** | **0.31** | **0.45** |
| *Chrysococcus rufescens* Klebs 1892 | 0.55 | 0.10 | 0.14 |
| *Goniochloris mutica* (A.Braun) Fott 1960 | 0.09 | 0.01 | 0.01 |
| *Goniochloris smithii* (Bourrelly) Fott 1960 | 0.13 | 0.20 | 0.30 |
| **Bacillariophyta** | **35.79** | **19.18** | **27.93** |
| *Aulacoseira granulata* (Ehrenberg) Simonsen 1979 | 2.21 | 3.65 | 5.32 |
| *Aulacoseira granulata* var. *angustissima* (Otto Müller) Simonsen 1979 | 13.25 | 2.35 | 3.42 |
| *Cocconeis placentula* Ehrenberg 1838 | 1.66 | 2.48 | 3.62 |
| *Cyclotella meneghiniana* Kützing 1844 | 3.31 | 7.02 | 10.23 |
| *Fragilaria acus* (Kützing) Lange-Bertalot in Krammer & Lange-Bertalot 2000 | 0.17 | 0.12 | 0.17 |
| *Navicula sp*. | 1.93 | 0.23 | 0.34 |
| *Nitzschia sp*. | 1.38 | 0.12 | 0.18 |
| *Stephanodiscus minutulus* (Kützing) Cleve & Möller 1882 | 11.87 | 3.19 | 4.65 |
| **Euglenophyta** | **5.70** | **18.30** | **26.66** |
| *Cryptoglena skujae* Marin & Melkonian in Marin et al. 2003 | 2.21 | 0.55 | 0.80 |
| *Euglena variabilis* G.A.Klebs 1883 | 0.26 | 0.84 | 1.23 |
| *Euglenaformis proxima* (P.A.Dangeard) M.S.Bennett & Triemer in Bennett et al. 2014 | 1.10 | 3.12 | 4.54 |
| *Lepocinclis oxyuris* (Schmarda) B.Marin & Melkonian in B.Marin et al. 2003 | 0.26 | 5.11 | 7.45 |
| *Lepocinclis playfairiana* (Deflandre) Deflandre 1932 | 0.21 | 2.56 | 3.72 |
| *Lepocinclis sp*. | 0.28 | 2.72 | 3.96 |
| *Monomorphina pyrum* (Ehrenberg) Mereschkowsky 1877 | 0.21 | 0.70 | 1.02 |
| *Strombomonas sp*. | 0.83 | 2.15 | 3.14 |
| *Trachelomonas volvocina* (Ehrenberg) Ehrenberg 1834 | 0.34 | 0.55 | 0.79 |
| **Dinophyta** | **0.32** | **1.60** | **2.33** |
| *Gymnodinium sp*. | 0.28 | 1.50 | 2.18 |
| *Peridinium sp*. | 0.04 | 0.11 | 0.16 |
| **Cryptophyta** | **0.26** | **0.61** | **0.88** |
| *Cryptomonas sp*. | 0.26 | 0.61 | 0.88 |
| **Bacteria** | **1.93** | **0.04** | **0.06** |
| *Planctomyces bekefii* Gimesi 1924 | 1.93 | 0.04 | 0.06 |
| **Total:** | **743.69** | **68.66** | **100.00** |
